# Preserving fertility in young women undergoing chemotherapy for early breast cancer; the Maastricht experience

**DOI:** 10.1007/s10549-020-05598-2

**Published:** 2020-03-31

**Authors:** Ingeborg J. H. Vriens, Elena M. ter Welle-Butalid, Maaike de Boer, Christine E. M. de Die-Smulders, Josien G. Derhaag, Sandra M. E. Geurts, Irene E. G. van Hellemond, Ernest J. T. Luiten, M. Wouter Dercksen, Bea M. D. Lemaire, Els R. M. van Haaren, Birgit E. P. J. Vriens, Agnes J. van de Wouw, Anne-marie M. G. H. van Riel, Sandra L. E. Janssen-Engelen, Marlène H. W. van de Poel, Ester E. M. Schepers-van der Sterren, Ron J. T. van Golde, Vivianne C. G. Tjan-Heijnen

**Affiliations:** 1 Division of Medical Oncology, Department of Internal Medicine, Maastricht University Medical Center, P.O. Box 5800, 6202 AZ Maastricht, The Netherlands; 2 GROW - School for Oncology and Developmental Biology, Maastricht University Medical Center, Maastricht, The Netherlands; 3 Department of Obstetrics and Gynaecology, Maastricht University Medical Center, Maastricht, The Netherlands; 4 Department of Clinical Genetics, Maastricht University Medical Center, Maastricht, The Netherlands; 5 Department of Surgery, Amphia Hospital, Breda, The Netherlands; 6 Department of Internal Medicine, Máxima Medical Center, Eindhoven, The Netherlands; 7 Department of Surgery, Elkerliek Hospital, Helmond, The Netherlands; 8 Department of Surgery, Zuyderland Medical Center, Heerlen, The Netherlands; 9 Department of Internal Medicine, Catharina Hospital, Eindhoven, The Netherlands; 10 Department of Internal Medicine, VieCuri Medical Center, Venlo, The Netherlands; 11 Elisabeth Twee Steden Hospital, Tilburg, The Netherlands; 12 Department of Surgery, St. Jans Hospital, Weert, The Netherlands; 13 Department of Internal Medicine, Laurentius Hospital, Roermond, The Netherlands; 14 Department of Surgery, St Anna Hospital, Geldrop, The Netherlands

**Keywords:** Fertility preservation, Breast cancer, Chemotherapy, Endocrine therapy, Ovarian function, Desire to have children

## Abstract

**Purpose:**

We assessed the uptake of fertility preservation (FP), recovery of ovarian function (OFR) after chemotherapy, live birth after breast cancer, and breast cancer outcomes in women with early-stage breast cancer.

**Methods:**

Women aged below 41 years and referred to our center for FP counseling between 2008 and 2015 were included. Data on patient and tumor characteristics, ovarian function, cryopreservation (embryo/oocyte) and transfer, live birth, and disease-free survival were collected. Kaplan–Meier analyses were performed for time-to-event analyses including competing risk analyses, and patients with versus without FP were compared using the logrank test.

**Results:**

Of 118 counseled women with a median age of 31 years (range 19–40), 34 (29%) chose FP. Women who chose FP had less often children, more often a male partner and more often favorable tumor characteristics. The 5-year OFR rate was 92% for the total group of counseled patients. In total, 26 women gave birth. The 5-year live birth rate was 27% for the total group of counseled patients. Only three women applied for transfer of their cryopreserved embryo(s), in two combined with preimplantation genetic diagnosis (PGD) because of BRCA1-mutation carrier ship. The 5-year disease-free survival rate was 91% versus 88%, for patients with versus without FP (*P* = 0.42).

**Conclusions:**

Remarkably, most women achieved OFR, probably related to the young age at diagnosis. Most pregnancies occurred spontaneously, two of three women applied for embryo transfer because of the opportunity to apply for PGD.

## Introduction

Breast cancer is the most commonly diagnosed malignancy in women, with approximately 12% of the women affected being younger than 40 years of age [1]. In these patients, (neo)adjuvant chemotherapy is frequently recommended, as younger age is an independent risk factor for an unfavorable outcome [2]. In patients at a reproductive age, chemotherapy may lead to premature ovarian insufficiency and subsequently to impaired fertility [3, 4]. With the currently used chemotherapy regimens the risk of permanent chemotherapy-induced ovarian function failure is on average 20% in patients below 40 years of age [3–5].

Considering the trend towards postponing the age of becoming pregnant, the number of women without children at diagnosis of breast cancer is increasing [6]. Moreover, at breast cancer diagnosis, it is generally recommended to postpone pregnancy for 2 years to allow resumption of adequate ovarian function and because of the relatively high risk of recurrence in this period [7].

Infertility following cancer treatment has a recognized negative impact on quality of life [8]. The prospect of loss of fertility has been reported to influence the choice of and adherence to a prescribed systemic treatment in over a quarter of cases [9–12]. International guidelines recommend that oncologists address the possibility of future infertility with patients with newly diagnosed cancer in their reproductive years [7, 13, 14]. Despite these guidelines, the likelihood of discussions regarding fertility preservation, among others, is influenced by unfamiliarity with fertility preservation processes, risks, and outcomes [15]. Patients should be referred as early as possible to specialists who can offer fertility preservation (FP). Initiation of a program with information about cancer treatment-related fertility issues and FP options has shown to significantly improve patient satisfaction [16, 17]. In our university hospital in the Southeast of the Netherlands, a FP program has been offered since 2008. The current study aimed to evaluate the uptake of FP in patients referred for counseling at breast cancer diagnosis and to assess fertility, live birth after breast cancer, and breast cancer outcome during follow-up.

## Methods

### Patients and study design

We performed a prospective cohort study in patients who visited the Maastricht University Medical Centre for counseling on FP in the years 2008–2015. Premenopausal patients aged under 41 years with stage I–III invasive breast cancer with an indication for adjuvant or neoadjuvant systemic treatment who were potentially interested in FP were referred. Patients were offered the option of embryo and/or oocyte cryopreservation. None of the patients received prophylactic GnRH analogs during chemotherapy. Our controlled ovarian stimulation protocol is according to the protocol described by Oktay et al*.* and von Wolff et al. [18, 19] According to the Netherlands Central Committee on Research Involving Human Subjects guidelines, this observational study is not subject to the Act on Medical Research Involving Human Subjects.

### Data collection

For each patient, the following characteristics were collected: date of first visit, age, gene mutation status and date, (male) partner at time of diagnosis, age and number of children at diagnosis if applicable, known infertility before treatment, primary tumor characteristics, type of local breast cancer treatment, chemotherapy, human epidermal growth factor receptor 2 (HER2)-targeted therapy and endocrine therapy if applicable, choice of FP procedure, number of oocytes retrieved, and number of embryos and oocytes frozen. During follow-up, we collected data on OFR, date of live birth, date of last update, first local, regional or distant breast cancer recurrence or the occurrence of a contralateral breast cancer, and survival status. Data collection from the medical files was last updated in 2018, therefore the follow-up duration was at least 2 years.

### Study objectives

We aimed to assess the uptake of FP in young women with early-stage breast cancer referred for counseling to the Maastricht University Medical Centre from 10 affiliated centers in the Southeast of the Netherlands in the years 2008–2015, and to assess the OFR-rate after end of chemotherapy, the live birth rate, and the disease-free survival rate since the date of counseling.

### Definitions

Premenopausal status at counseling was based on a history of regular menstruation. In patients using oral contraceptives, we presumed a premenopausal status based on a regular menstrual cycle before oral contraceptive use and the young age at counseling [20]. Furthermore, before the start of hyperstimulation, we performed a vaginal ultrasound to count the antral follicles and measured Anti-Mullerian Hormone to predict the ovarian reserve. Uptake of FP was defined by the number of women who underwent FP as numerator and the number of counseled women as denominator. Premenopausal status after chemotherapy (OFR) was based on menstrual cycles and/or premenopausal lab values. History and FSH/17-ß-estradiol assessments were locally performed at the end of chemotherapy and 3-monthly thereafter. Time to OFR was defined as the interval from the end of chemotherapy to the date of premenopausal status confirmation (set at 0.1 months if present at the end of chemotherapy). Hence, OFR is a composite endpoint consisting of both recovery and maintenance of ovarian function during chemotherapy, as one may consider it equally relevant from a patient perspective when estimating on forehand the risk of infertility. The rate of live birth was defined as the percentage of women that gave birth to one or more babies excluding still birth and miscarriage. The time to live birth was defined as the interval from the date of counseling to the first live birth. The mode by which the pregnancy leading to live birth was achieved was categorized as either spontaneous, by use of earlier cryopreserved embryos or oocytes, or by fertility treatments. The period of disease-free survival was defined as the interval from the date of counseling to ipsilateral invasive breast tumor recurrence, regional breast cancer recurrence, distant recurrence, contralateral breast cancer, second primary non-breast invasive cancer, or death attributable to any cause, whichever occurred first [21].

### Statistical analysis

Baseline characteristics of the FP group and the non-FP group were compared using independent samples Student *t* tests for normally distributed continuous variables, the Mann–Whitney-U test for skewed continuous variables, and the chi-square test for categorical variables. Kaplan–Meier analyses were performed for OFR (one minus Kaplan–Meier estimator) and disease-free survival, for disease-free survival comparing the FP group and the non-FP group by using the logrank test. Cumulative incidence of live birth was determined using competing risk analyses. For the endpoint of OFR, patients were censored at the date of ovariectomy, start of gonadotropin-releasing hormone treatment in the absence of OFR, recurrent disease or last follow-up. In patients with OFR for whom the exact date of OFR was not known (*n* = 13), we assumed the group median as time to OFR to prevent exclusion of these patients and an underestimation of the OFR-rate. For the endpoint of live birth rate, patients were censored at the end of follow-up. The occurrence of a disease-free survival event and ovariectomy, both hindering the observation of live birth, were therefore considered as competing risks. All statistical analyses were performed with SPSS 22.0 and STATA 14.1. A *P* value of < 0.05 was considered statistically significant.

## Results

### Patient characteristics

**Fig. 1:**
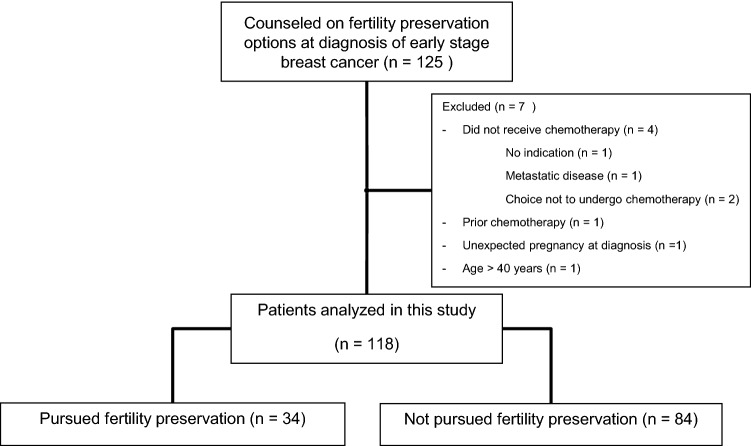
Flow chart about the patient selection

In the period 2008–2015, 125 women with the diagnosis of early breast cancer were referred for FP counseling. After excluding seven patients because of several reasons, 118 patients were analyzed in this study (Fig. 1).

Patient, tumor, and treatment characteristics are shown in Table 1. The median age of the counseled women was 31 years (range 19–40). At diagnosis, 75% of patients had a male partner and 31% had one or more children. Of all patients, 84% were analyzed for germline mutations. In 19% of patients, a germline mutation was present, corresponding with 23% of tested patients. The median tumor size was 22 mm (range 7–100) with slightly more than half of the patients having lymph node-negative disease. Almost two-third of the patients had a grade III tumor. The hormone receptor status was positive in 53% and the HER2 status was positive in 25%. The median follow-up in the FP group was 52 months (95% confidence interval (CI) 48–58), the median follow-up in the non-FP group was 51 months (95% CI 42–63).

**Table 1:** Baseline demographic and clinical characteristics, *N* (%)

	All *N* = 118	FP group *N* = 34	Non FP group *N* = 84	FP vs non FP *P* value
Age at diagnosis (years)				0.80
Mean	31	31	31	
Range	19–40	23–40	19–40	
Male partner at the time of breast cancer diagnosis				0.06
Yes	89 (75)	30 (88)	59 (70)	
No	29 (25)	4 (12)	25 (30)	
Children at the time of breast cancer diagnosis				0.03
No	82 (69)	29 (85)	53 (63)	
Yes	36 (31)	5 (15)	31 (37)	
Infertility before cancer diagnosis				0.28
Yes	9 (8)	4 (12)	5 (6)	
No	109 (92)	30 (88)	79 (94)	
Germline mutation carrier				0.86
Positive test	23 (19)^a^	7 (21)	16 (19)	
Negative test	77 (65)	21 (62)	56 (67)	
No test	18 (15)	6 (18)	12 (14)	
Tumor size (mm)				0.04
Median	22	19	25	
Range	7–100	9–53	7–100	
Lymph node status				0.26
N0/N0(i+)	65 (55)	23 (68)	42 (50)	
N1mi/N1a	37 (44)	9 (26)	28 (33)	
N2/N3	14 (12)	2 (6)	12 (14)	
Nx	2 (2)	0 (0)	2 (2)	
Tumor grade				0.52
1	9 (8)	3 (9)	6 (7)	
2	31 (26)	7 (21)	24 (29)	
3	75 (64)	24 (71)	51 (61)	
Unknown	3 (3)	0 (0)	3 (4)	
Histology				0.18
Ductal	110 (93)	34 (100)	76 (90)	
Lobular	4 (3)	0 (0)	4 (5)	
Medullar	4 (3)	0 (0)	4 (5)	
Hormone receptor status				0.07
Positive	63 (53)	23 (68)	40 (48)	
Negative	55 (47)	11 (32)	44 (52)	
HER2 status				0.48
Positive	29 (25)	10 (29)	19 (23)	
Negative	89 (75)	24 (71)	65 (77)	
Local therapy				0.54
Breast conserving	34 (29)	10 (38)	34 (40)	
Unilateral mastectomy	39 (33)	14 (41)	25 (30)	
Bilateral mastectomy	34 (29)	10 (29)	24 (29)	
Unknown	1 (1)	0 (0)	1 (1)	
Chemotherapy^b^				0.04
Second generation	9 (8)	2 (6)	7 (8)	
Third generation	108 (92)	32 (94)	76 (90)	
Other	1 (1)	0 (0)	1 (1)	
HER2-targeted therapy				0.48
Yes	29 (25)	10 (29)	19 (23)	
No	89 (75)	24 (71)	65 (77)	
Endocrine therapy				0.07
Yes	60 (51)	22 (65)	38 (45)	
Tamoxifen only	11 (9)	2 (6)	9 (11)	
Tamoxifen + OFS^c^	46 (39)	19 (56)	27 (32)	
Tamoxifen followed by aromatase inhibitor + OFS	3 (3)	1 (3)	2 (2)	
No	58 (49)	12 (35)	46 (55)	

^a^BRCA1 gene mutation detected in 15 patients, BRCA2 gene mutation in 4 patients, CHECK2 mutation in 3 patients, PTEN mutation in 1 patient

^b^Second-generation chemotherapy consisted of six 3-weekly cycles of FEC (5-fluorouracil, epirubicin, cyclophosphamide, 500, 100, and 500 mg/m^2^, respectively). Third-generation chemotherapy consisted of six 3-weekly cycles TAC (docetaxel, doxorubicin, and cyclophosphamide, 75, 50, and 500 mg/m^2^, respectively) or eight 3-weekly cycles AC-T (four cycles of doxorubicin and cyclophosphamide followed by four 3-weekly cycles of docetaxel or paclitaxel (AC-T; 60, 600, and 100 mg/m^2^ or 80 mg/2 weekly, respectively), whether or not in combination with trastuzumab (2 mg/kg). One other patient was treated with carboplatin (AUC2)/paclitaxel(80 mg/m^2^)/trastuzumab (2 mg/kg). So, all women received an alkylating-based regimen

^c^OFS, ovarian function suppression (chemotherapy-induced ovarian function failure or LhRHa or oophorectomy)

### Uptake of fertility preservation

Thirty-four (29%) women underwent FP before the start of chemotherapy. Patients who underwent FP had less often children (85% versus 63%, *P* = *0.03*), more often a male partner (88% versus 70%, *P* = *0.06*), and more often smaller tumors (19 mm versus 25 mm, *P* = *0.04)* compared with those who did not undergo FP (Table 1).

In 33 of the 34 (97%) patients who pursued FP, embryos or oocytes could be cryopreserved. In one patient only one oocyte was obtained, which could not be fertilized. In 25 patients embryos were frozen and in seven oocytes. One patient chose to preserve both embryos and oocytes. Per patient a median of four embryos (range 1–17) and seven oocytes (range 2–13) were cryopreserved. In three patients, two cycles of oocyte retrieval were necessary, the other patients underwent one cycle.

### Ovarian function

**Fig. 2:**
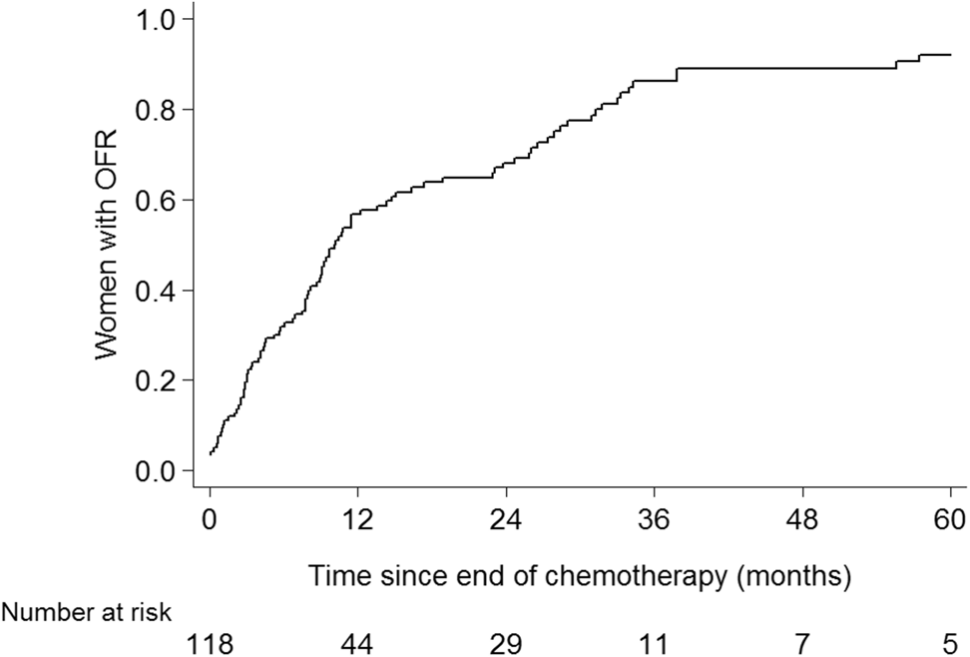
Rate of ovarian function recovery. *OFR* ovarian function recovery

In all but four patients menses was stopped during chemotherapy. Median time to OFR was 9 months (range 0–83 months). The 2-year OFR-rate was 68% (95% CI 59–77%). The 5-year OFR rate was 92% for the total group of counseled patients (Fig. 2).

### Live birth

**Fig. 3:**
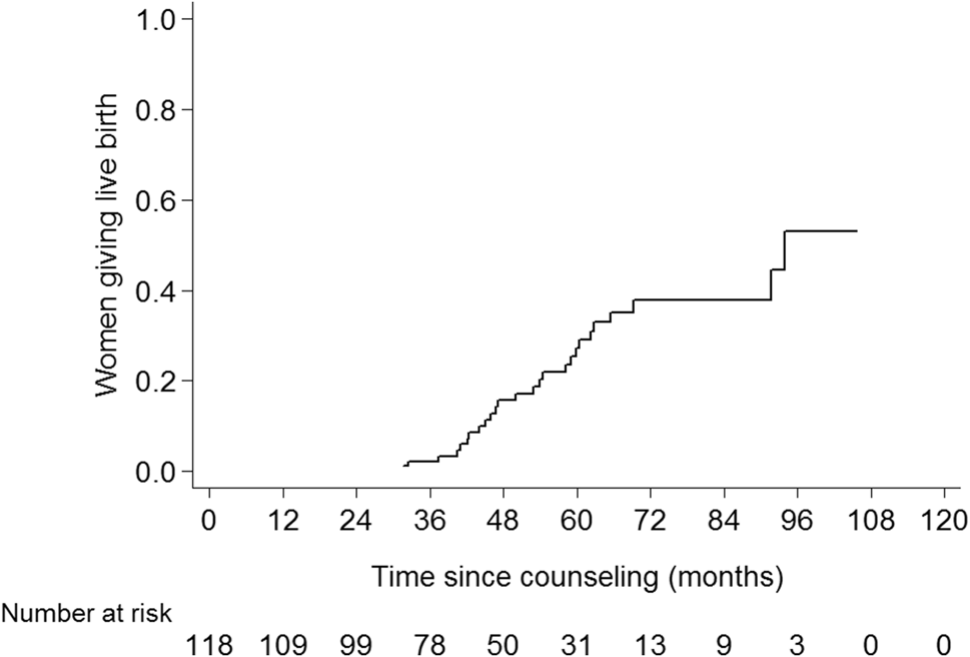
Rate of live birth

Live birth occurred in 26 women. All pregnancies occurred at least 2 years after the diagnosis of breast cancer. The 5-year live birth rate was 27% (95% CI 17–38%, Fig. 3). Of the 26 women giving live birth, eleven had estrogen receptor-positive disease.

Ten women in the FP group gave birth to twelve babies. Eleven babies were healthy, one had a congenital anomaly (M. Hirschsprung).

Only three women applied for transfer of cryopreserved embryos. Two of these women asked for PGD of the frozen embryos because they had a germline mutation in the BRCA1 gene, although both had OFR. Both women became pregnant after transfer of an embryo without the BRCA1 mutation. One other woman did not conceive after thawing of 10 and transfer of 8 embryos. Also, her attempts to achieve a spontaneous pregnancy were not successful. Another patient was supported by intrauterine insemination (IUI) to become pregnant. The other nine pregnancies in this group were spontaneous pregnancies.

Sixteen women in the non-FP group gave birth to twenty healthy babies. All but one pregnancy in this group were spontaneous. One woman conceived via IVF after breast cancer treatment. One woman did not become pregnant despite OFR and the use of IVF and IUI.

### Disease-free survival

**Fig. 4:**
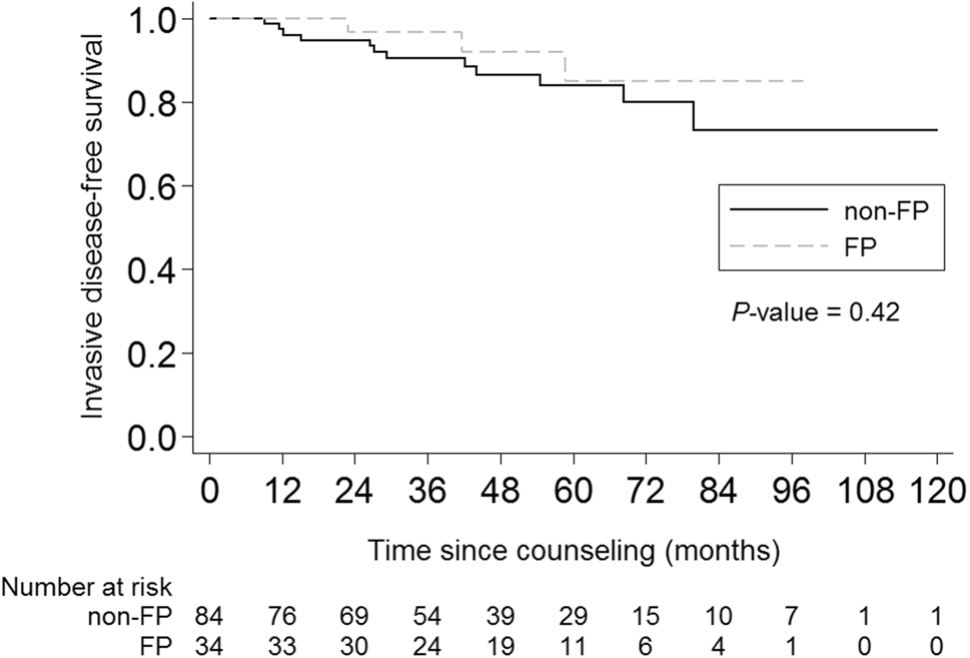
Invasive disease‐free survival rate. *Non FP* women who have not pursued fertility preservation treatment, *FP* women who did pursue fertility preservation treatment

Disease events were detected in fifteen patients. The 5-year disease-free survival rate was 85%. In the FP group, two patients developed distant metastases, of whom one is deceased, one patient had a loco-regional recurrence. In the non-FP group (*n* = 84), twelve women developed distant metastases, of whom four were deceased. For women in the FP group, the 5-year disease-free survival rate was 85% and for women in the non-FP group, the rate was 84% (logrank *P* = *0.42,* Fig. 4).

## Discussion

Twenty-nine percent of 118 young women with early breast cancer diagnosis elected FP after counseling. Predictive factors for choosing FP were childlessness at the time of diagnosis, having a male partner and smaller tumors. After a median follow-up of 52 months, only three of 34 couples used their frozen embryos in an attempt to achieve live birth. Interestingly, all three had OFR, and two of these used the embryos for PGD because of a BRCA1-mutation carriership. Also, in the total group, the 5-year OFR was more than 90%. Twenty-six mothers gave birth to thirty-two babies. The 5-year live birth rate was 27%.

Before initiating systemic treatment for breast cancer at a young age, the option of FP by cryopreservation of embryos of oocytes should be considered [13]. In our study, 29% of referred women underwent FP. Of note, we only counseled women who were potentially interested in FP. Other studies reported FP rates varying from 9 to 58%, with the lower rates seen in studies in unselected young women [9, 12, 22–26]. Based on the Dutch Cancer Registry database, we estimate that approximately 20% of women < 41 years and 35% of women < 35 years diagnosed with breast cancer wished referral for counseling. Remarkably, many patients in our study stated at the start of counseling that they tended not to opt for FP, even though they were not yet fully informed. However, counseling on the possible benefits and harms of chemotherapy including impact on fertility and the option of FP are important for the patient to make a well-informed decision before the initiation of chemotherapy in order to prevent regrets afterwards [17].

To date, the available data on the transfer of cryopreserved oocytes or embryos after breast cancer therapy are limited [18, 25, 27–29]. Embryo or oocyte transfer rates of 6—25% are reported [18, 25, 27–29]. In our study, only three women returned for embryo transfer, with a relatively high rate of spontaneous pregnancies. In the earlier reported studies, there is no information on OFR rates. Our current and former studies have shown high OFR rates in women < 40 years [30]. A quarter of patients had OFR between 2 and 5 years after counseling. When live birth is strived for too soon, the ovarian function may yet not have had time to recover.

Though FP in breast cancer patients is feasible and safe, the individual risk estimation of premature ovarian insufficiency should be part of the counseling procedure. Previously, we reported that with the currently used chemotherapy schedules, age is still the most important factor predicting OFR after chemotherapy [30, 31]. Petrek et al. showed that in women younger than 35 years of age, the long-term (more than 3 years after diagnosis) incidence of amenorrhea was similar to that of women who had not received chemotherapy (nearly 10%) [32]. In our current study, in patients < 30 years, at least 93% had OFR. Given these data, very young women may decide not to undergo a FP procedure. However, after completion of prolonged adjuvant endocrine therapy, irrespective of prior use of chemotherapy, natural aging may have caused loss of ovarian reserve and thus infertility, despite the presence of OFR. Although interruption of adjuvant endocrine therapy is used in daily practice, its safety is still under investigation in the POSITIVE trial (ClinicalTrials.gov Identifier: NCT02308085) [33]. For patients with BRCA 1/2-gene mutations, another argument for embryo or oocyte cryopreservation can be the option of PGD and the possibly shorter reproductive life span in these patients [34–37].

Luteinizing hormone releasing hormone analogs (LHRHa) are being used in premenopausal women to protect the ovarian function during chemotherapy [38–40]. Of note, the primary aim of the studies was to reduce the occurrence of premature menopause instead of improving live birth rates. As a result, the included population may not be appropriate to address the live birth rate. The added value of LHRHa in terms of live birth rate in younger women could thus be less than assumed from these studies. The POEMS trial is the only randomized controlled trial that reported pregnancies as preplanned endpoint and also reported live births. The live birth rate was 7% [38, 39]. A meta-analysis showed a 6% pregnancy rate in women undergoing chemotherapy alone [40]. The live birth rates were not reported. These rates are significantly lower as compared to the 5-year live birth rate in our study (27%), whereas none of the patients in our cohort received prophylactic LHRHa use. A possible explanation is the lower median age of the women in our study (31 years) as compared with the median age in the meta-analysis (39 years) [32, 41].

FP after diagnosis of breast cancer is considered a safe option [24, 42]. Even in women with estrogen receptor-positive breast tumors, small retrospective studies have shown no detrimental effect of FP with the estrogen-positive tumor in situ [28, 42–45]. Stimulation protocols that add letrozole or tamoxifen have been successfully implemented and keep serum estradiol close to physiologic levels during the cycle [43, 46, 47]. Live birth after breast cancer, even in endocrine sensitive tumors, is currently considered safe [48–51]. In our study, fifteen women had recurrent disease, five of them within 2 years after diagnosis. None of these patients had given live birth after the diagnosis of breast cancer.

Apart from the relative short follow-up time, a limitation of our study is that we do not have data for actual and persistent desire to have children during follow-up. Women may have tried to conceive as soon as the ovarian function recovered, or they might regret the choice of not having cryopreserved embryos of oocytes. Since pregnancy attempts were not prospectively registered, it could be that during follow-up, their responsible oncologist paid no active attention to it. Moreover, we did not collect information on the maintenance of their relationship. Hence, we are not sure whether the previous choice to cryopreserve embryos instead of oocytes may have been a barrier for some to achieve a pregnancy. However, the observation that a substantial number of patients gave live birth to one or more children is reassuring.

In conclusion, in our cross-sectional study, nearly one-third of the counseled women chose to undergo a procedure for FP. About a quarter of women gave birth within 5 years after counseling, in nearly all spontaneously. Only three women used their frozen embryos to achieve a live birth: two applied for PGD of their cryopreserved embryos because of a BRCA1 mutation, resulting in the birth of two healthy children. The third woman did not become pregnant after transfer of embryos. More research in this field is required, as fulfillment of the desire to have children is clearly related to the quality of life.
